# Methyl Jasmonate Protects the PS II System by Maintaining the Stability of Chloroplast D1 Protein and Accelerating Enzymatic Antioxidants in Heat-Stressed Wheat Plants

**DOI:** 10.3390/antiox10081216

**Published:** 2021-07-28

**Authors:** Mehar Fatma, Noushina Iqbal, Zebus Sehar, Mohammed Nasser Alyemeni, Prashant Kaushik, Nafees A. Khan, Parvaiz Ahmad

**Affiliations:** 1 Plant Physiology and Biochemistry Laboratory, Department of Botany, Aligarh Muslim University, Aligarh 202002, India; meharfatma30@gmail.com (M.F.); seharzebus5779@gmail.com (Z.S.); 2 Department of Botany, School of Chemical and Life Sciences, Jamia Hamdard, New Delhi 110062, India; naushina.iqbal@gmail.com; 3 Botany and Microbiology Department, College of Science, King Saud University, Riyadh 11451, Saudi Arabia; mnalyemeni@gmail.com; 4 Kikugawa Research Station, Yokohama Ueki, 2265, Kamo, Kikugawa City, Shizuoka 439-0031, Japan; kaushik.prashant@yokohamaueki.co.jp

**Keywords:** antioxidant, methyl jasmonate, photosystem

## Abstract

The application of 10 µM methyl jasmonate (MeJA) for the protection of wheat (*Triticum aestivum* L.) photosystem II (PS II) against heat stress (HS) was studied. Heat stress was induced at 42 °C to established plants, which were then recovered at 25 °C and monitored during their growth for the study duration. Application of MeJA resulted in increased enzymatic antioxidant activity that reduced the content of hydrogen peroxide (H_2_O_2_) and thiobarbituric acid reactive substances (TBARS) and enhanced the photosynthetic efficiency. Exogenous MeJA had a beneficial effect on chlorophyll fluorescence under HS and enhanced the pigment system (PS) II system, as observed in a JIP-test, a new tool for chlorophyll fluorescence induction curve. Exogenous MeJA improved the quantum yield of electron transport (ET_o_/CS) as well as electron transport flux for each reaction center (ET_0_/RC). However, the specific energy fluxes per reaction center (RC), i.e., TR_0_/RC (trapping) and DI_0_/RC (dissipation), were reduced by MeJA. These results indicate that MeJA affects the efficiency of PS II by stabilizing the D1 protein, increasing its abundance, and enhancing the expression of the *psb*A and *psb*B genes under HS, which encode proteins of the PS II core RC complex. Thus, MeJA is a potential tool to protect PS II and D1 protein in wheat plants under HS and to accelerate the recovery of the photosynthetic capacity.

## 1. Introduction

Wheat is the main cereal crop belonging to the Poaceae family, which contributes 30% and 50% to the world grain production and grain trade, respectively [1]. Its quality and yield are directly associated with countrywide food security [2]. However, wheat plants often suffer heat stress, which affects their quality and utilization worldwide. India and China alone are expected to show a decrease in wheat yield by 8% and 3%, respectively, due to the rise in worldwide mean temperature by 1 °C. The impact of high temperature on the growth of plants is due to the excess output of reactive oxygen species (ROS) that damage the photosynthetic apparatus of plants by decreasing the rate of photosynthetic electron transport, inactivating the pigment system (PS) II center, degrading pigments and proteins, and eventually decreasing the yield [3]. ROS produced in chloroplasts as a result of abiotic stress hinder the synthesis of D1 protein, a component of the PS II complex [4]. D1 is the main subunit of the complex which not only regulates the binding of cofactors, but also maintains the structure of the reaction center of PS II, engaged in essential charge division and electron transport [5,6]. The D1 protein encoded by the chloroplast gene *psbA* is the main target of damage under the environmental stress conditions. It was reported that quick repair of D1 protein is essential for the efficient recovery of PS II [2,7]. Plants contain established restoration mechanisms to check damage to PS II, but little is known about the mechanisms of protection of the PS II system under heat stress.

The adaptive reaction of plants to oxidative stress consists in the rise of the activity of antioxidants enzymes [8,9,10]. Remarkably, several studies reported the association between antioxidant activity, heat tolerance, and plant hormones [3,11,12,13]. Among plant hormones, jasmonates promotes protection from abiotic stresses. In particular, methyl jasmonate (MeJA) has shown promising effects in the defense of plants under diverse abiotic stresses [8,14,15]. Previous studies established that MeJA alleviated oxidative stress by improving antioxidant enzymes activities [8,16]. Exogenous application of MeJA protected *Arabidopsis thaliana* and *Citrus reticulata × Citrus sinensis* plants against the inhibitory effect on chlorophyll (Chl) induced by copper and cadmium and promoted photosynthetic activity [17,18]. Attaran et al. [19] reported that RNA sequencing and chlorophyll fluorescence imaging were important in studying jasmonic acid effect on photosynthetic capacity, growth, and gene expression in *Arabidopsis*. It has been reported that D1 protein turnover is more rapid than that of any other protein in the thylakoid membrane under light illumination [2]. This feature exposes PS II to photoinduced damage, causing photoinhibition and reduction in photosynthetic efficiency. Henceforth, D1 protein degradation, synthesis, and reinsertion into the PS II core complex represent an important aspect of PS II dynamics [5,20].

The regulatory mechanisms by which MeJA protects the PS II system, particularly through stabilization of D1 protein and regulation of relevant gene expression in wheat leaves under heat stress have not been investigated. Therefore, the purpose of the present study was to examine the effect of MeJA on the protection of the PS II complex, chlorophyll fluorescence, activity of enzymatic antioxidants, D1 protein abundance, and photosynthetic gene expression in wheat leaves under heat stress. Additionally, this study also investigated how MeJA affected plant growth and CO_2_ assimilation in the leaves of heat-stressed wheat plants. Our findings demonstrated that MeJA application can protect the PS II complex from heat stress-induced damage by increasing the levels of enzymatic antioxidants and accelerating the stability of D1 protein in wheat leaves.

## 2. Materials and Methods

Wheat (*Triticum aestivum* L.) cultivar HD 2329 (winter wheat) seeds were collected from the Indian Agricultural Research Institute (IARI), New Delhi, surface-sterilized with 0.01% HgCl_2_ followed by 3–4 frequent washings with deionized water, and then seeded in 14 cm diameter pots (14 cm diagonally to the top and 18 cm toward the bottom). The pots were filled with 4 kg sterilized acid-washed fine sand with a particle size from 125 µm to 250 µm and pH of about 7.0. The sand was first purified with a mixture of 17% *w*/*v* hydrochloric acid and 1% oxalic acid in a sand digester (electrode boiler) for 6 h. After that, the sand was washed with deionized water before being transferred to the pots. The purification of sand was performed by the following the method of Hewitt et al. [21]. A plant growth chamber (Khera KI-261, Khera Instruments Pvt. Ltd., New Delhi, India) set with day/night temperatures of 25/17 ± 3 °C, 12 h photoperiod (Photosynthetically Active Radiation; PAR; 350 µmol m^−2^ s^−1^), and 65 ± 5% relative humidity was used. Three plants were kept in each pot and were given 300 mL Hoagland’s solution (full strength) on alternate days. In the experimentation, the heat stress treatment was applied to 3–4 leaves at emergence stage by exposing the plants to 42 °C every day for 6 h for 10 DAS (days after sowing). After that, the plants were recovered at 25 °C (no stress; optimum temperature) and were grown for 5 more days. The experimentation continued for 30 days. Plants were supplied with Hoagland’s nutrient solution alternatively in the morning. The control group of plants for each pot was kept for 30 days at 25 °C throughout the experimental duration. The plants were sprayed with 5, 10, and 20 µM MeJA (Sigma Aldrich, St. Louis, MO, USA) in the absence (25 °C) or presence (42 °C) of heat stress at 15 DAS with a hand sprayer. Plants were sampled for the determination of oxidative stress, growth, and photosynthetic characteristics 30 DAS. A detailed examination on the effects of 10 µM MeJA (selected on the basis of the concentrations used in the experiment) in the alleviation of heat stress was performed. The MeJA solution and control distilled water were sprayed along with 0.5% surfactant Teepol. The randomized complete block design was adopted for the treatments, and the number of replicates for every treatment was four (*n* = 4). At the vegetative growth stage, the leaves were sampled and preserved (−80 °C) for physio-biochemical analyses, to examine antioxidant enzyme activities and photosynthetic pigments, Western blotting, and quantitative reverse transcription polymerase chain reaction (qRT-PCR) quantification. Harvesting was done 30 DAS, and it was assured that leaves were taken at a similar stage for the measurements.

### 2.1. Measurement of Reactive Oxygen Species Content and Lipid Peroxidation

Superoxide radicals (O_2_^−^) content was estimated with the method of Bu et al. [22] and Lang et al. [14] with slight changes. Fresh leaf tissues (200 mg) were treated with 1 mL hydroxylamine hydrochloride for 1 h, after which, 1 mL each of α-naphthylamine and *p*-aminobenzene sulfonic acid was added, and the solution was kept at 25 °C for 20 min. The absorbance of the solution was recorded at 530 nm, and the O_2_^−^content was calculated from a calibration curve using NaNO_2_ as the standard. Determination of hydrogen peroxide (H_2_O_2_) was performed by the technique of Okuda et al. [23]. Fresh leaf tissues (200 mg) were ground in ice-cold 200 mM perchloric acid, followed by centrifugation at 1200× *g* for 10 min. After centrifugation, perchloric acid in the supernatant was neutralized with 4 M KOH, and the insoluble potassium perchlorate was eliminated by further centrifugation at 500× *g* for 3 min. The reaction mixture contained 1 mL of the eluate, 400 µL of 12.5 mM 3-(dimethylamino) benzoic acid in 0.375 M phosphate buffer (pH 6.5), 80 µL of 3-methyl-2-benzothiazoline hydrazone, and 20 µL of peroxidase (0.25 unit) in a final volume of 1.5 mL. The reaction was started by the addition of peroxidase at 25 °C, and the increase in absorbance was recorded at 590 nm.

Lipid peroxidation was estimated by assessing thiobarbituric reactive substances (TBARS), as per Dhindsa et al. [24]. Fresh leaf tissues were ground in 0.25% 2-thiobarbituric acid (TBA) in 10% trichloroacetic acid (TCA) using mortar and pestle. After heating at 95 °C for 30 min, the mixture was quickly cooled in an ice bath and centrifuged at 10,000× *g* for 10 min. To 1 mL aliquot of the supernatant, 4 mL 20% TCA containing 5% TBA was added. The absorbance of the supernatant was read at 532 nm and corrected for nonspecific turbidity by subtracting the absorbance of the same at 600 nm. The content of TBARS was calculated using the extinction coefficient (155 mM^−1^ cm^−1^).

### 2.2. Assay of Antioxidant Enzymes Activities

The activity of enzymatic antioxidant catalase (CAT) was estimated following the procedure of Aebi [25] by monitoring the disappearance of H_2_O_2_ at 240 nm. The activity was calculated by using the extinction coefficient of 0.036 mM^−1^ cm^−1^. One unit of enzyme is the amount necessary to decompose 1 µmol of H_2_O_2_ per min at 25 °C.

The activity of ascorbate peroxidase (APX) was calculated according to Foyer and Halliwell [26] by the decrease in absorbance of ascorbate at 290 nm. The assay mixture contained phosphate buffer (50 mM, pH 7.0), 0.1 mM EDTA, 0.5 mM ascorbate, 0.1 mM H_2_O_2_, and the enzyme extract. The activity of APX was calculated by using the extinction coefficient of 2.8 mM^−1^ cm^−1^. One unit of the enzyme is the amount necessary to decompose 1 µmol of substrate per min at 25 °C.

The activity of glutathione reductase (GR) was determined according to Nakano and Asada [27] by monitoring the glutathione-dependent oxidation of nicotinamide adenine dinucleotide phosphate (NADPH) at 340 nm. The assay mixture contained phosphate buffer (25 mM, pH 7.8), 0.5 mM oxidized glutathione (GSSG), 0.2 mM NADPH, and the enzyme extract. The activity of GR was calculated by using the extinction coefficient of 6.2 mM^−1^ cm^−1^. One unit of enzyme is the amount necessary to decompose 1 µmol of NADPH per min at 25 °C. The details of the method were described before by Fatma et al. [28,29,30].

Superoxide dismutase (SOD) activity was assayed by the method of Giannopolitis and Ries [31] also used by Beyer and Fridovich [32], with slight alterations, by monitoring the inhibition of photochemical reduction of nitro blue tetrazolium (NBT). Five mL of the reaction mixture containing 5.0 mM (4-(2-hydroxyethyl)-1-piperazineethanesulfonic acid; HEPES) having pH 7.6, 0.1 mM ethylene diaminetetraacetic acid (EDTA), 50 mM Na_2_CO_3_ (pH 10.0), 13 mM methionine, 0.025% (*v*/*v*) triton X-100, 63 μmol NBT, 1.3 μmol riboflavin, and the enzyme extract was illuminated for 15 min (360 μmol m^−2^ s^−1^). A control set of experiments was also illuminated for correcting the background absorbance. A unit of SOD was defined as the amount of enzyme that inhibited NBT reduction by 50% at 560 nm.

### 2.3. Protein and Pigment Analysis

Fresh leaves (100 mg) were placed in 90% ammoniacal acetone for the determination of pigments and analyzed as described by Porra et al. [33]. The carotenoid content was calculated according to the method by Wellburn and Lichtenthaler [34], and Bradford [35] method was used for the estimation of total protein content, using bovine serum albumin as a standard.

### 2.4. Chlorophyll a Fluorescence Measurement

A Junior-PAM chlorophyll fluorometer (Heinz Walz GmbH, Eichenring, Effeltrich, Germany) was employed for the determination of chlorophyll fluorescence at room temperature. Leaves (mostly from the top of the plants) were adapted to the dark for 20 min before the fluorescence measurements [36]. Maximum (Fm) and minimal fluorescence (Fo) were studied in dark-adapted leaves with a light intensity of 131 µmol m^−2^ s ^−1^ (low beam intensity). In the light-adapted state, maximum fluorescence (Fm’) and minimal fluorescence (Fo’) were computed in similar leaves using a saturating light intensity at 830 µmol m^−2^ s^−1^, well balanced with steady-state fluorescence (Fs). The fluorescence values Fm-Fo and Fm’-Fo’ were used for the calculation of variable fluorescence (Fv and Fv’). The intrinsic efficiency of the PS II system indicated by Fv’/Fm’ and the actual PS II efficiency indicated by Fm’-Fs/Fm’ were calculated. Moreover, nonphotochemical (NPQ) and photochemical quenching (qP) were evaluated by using fluorescence parameters estimated in both light- and dark-adapted conditions [37].

### 2.5. Analysis of OJIP Chlorophyll a Fluroscense Transient 

Chlorophyll fluorescence transient was calculated in leaves dark-adapted for 20 min at room temperature by a Handy PEA (Plant Efficiency Analyzer, Hansatech Instruments, King’s Lynn, Norfolk, UK). Chlorophyll fluorescence transients were computed using light excitation (at 650 nm) at high intensity (3500 µmol photons m^−2^ s ^−1^) up to 2 s, with the help of an array of 3 LEDs. The OJIP transient data were first described by Strasser et al. [38]. O (beginning) was the initial minimum fluorescence (measured), which was accompanied by an increase toward the J level (2 ms), an inflection I (30 ms), and finally the peak *p* (300 ms). The OJIP transient data were calculated by the JIP test as defined by Govindjee [39], Stirbet, et al. [40] and Chen et al. [41]. The parameters labelled as Fv/Fo (variable to minimal fluorescence) which reflect the water-splitting complex activity taking place on the donor side of PS II, Fv/Fm (variable to maximal fluorescence), RC/ABS (ratio of reaction centers (RCs) to PS II antenna absorption), performance index (PI), and Area, i.e., the area above the chlorophyll fluorescence curve among Fo and Fm, which estimates the plastoquinone pool size, were analyzed by Biolyzer 4 HP (Version 4.0.30.03.02, Bioenergetics Laboratory, University of Geneva, Geneva, Switzerland) and PEA plus software (Version 1.02, Hansatech Instruments, King’s Lynn, Norfolk, UK). PI indicates a grouping of three self-regulating efficient phases of photosynthesis, i.e., RCs, indicating chlorophyll density, trapping of excitation energy, change of excitation energy toward the electron transport according to a particular many-parametric expression [42], and was determined as PI_ABS_: RC/ABS * PHIo/(1 − PHIo) * PSIo/(1 − PSIo), where RC/ABS is the RCs density for each PS II antenna chlorophyll, PHI is the quantity of excitons trapped per photon absorbed, and PSIo is the possibility of an electron transfer for the entire the way to PS I [38]. The fluorescence transients were normalized to Fo (at O level) for the calculation the OJIP data from different treatments. Additionally, the JIP test was performed for the parameters of the OJIP transient to quantify PS II behavior, which was established on the energy fluxes and yields. Therefore, for (A), the specific energy fluxes (per RC) as (i) Trapping (TR_0_/RC); (ii) Dissipation (DI_0_/RC), and (iii) Electron transport (ET_0_/RC) and for (B), the yields as quantum yield of electron transport (ET_o_/CS) were analyzed.

### 2.6. Photosynthetic and Growth Parameters

Photosynthetic efficiency determined as net photosynthesis (*P*n), intercellular CO_2_ concentration (Ci) and stomatal conductance (gs), was determined in the entirely developed uppermost plants leaves for each treatment by an Infrared Gas Analyzer (model CID-340; Bio-Science, Camas, WA, USA). The measurements were completed at 350 µmol photons m^−2^ s^−1^ with a CO_2_ concentration of 370 ± 5 µmol mol^−1^ and relative humidity of 65 ± 5% at temperature 25 °C.

Estimation of leaf area was done by a leaf area meter (model LA-211; Systronics, New Delhi, India), while plant fresh weight was measured by using a digital scale (Sartorius, Göttingen, Germany). For measuring dry weight, the plants were dried in an oven at 80 °C till constant weight.

### 2.7. Western Blot Analysis

Fresh leaves (1 g) were crushed in liquid nitrogen to obtain a fine powder for the extraction of the thylakoid protein, as described by Zhao et al. [2] with slight modifications. For Western blot assay, Chl was measured in the thylakoid membrane extracts, and thylakoid proteins (20 µg) having equal Chl were separated by 15% sodium dodecyl sulphate–polyacrylamide gel electrophoresis (SDS-PAGE) using 6 M urea and afterward blotted on nitrocellulose membranes [43]. D1 protein immunodetection was conducted using a polyclonal antibody raised against it according to a previous report [2,44]. The membranes were incubated for 2 h with primary anti-*psb*A antibodies (PhytoAB, San Francisco, CA, USA) and then incubated again with horseradish peroxidase-conjugated anti-rabbit IgG antibodies (PhytoAB, San Francisco, CA, USA) for 2 h. The housekeeping α-tubulin protein was used as an internal loading control for normalization. Pierce ECL plus substrate (Thermo Fisher Scientific, New Delhi, India) was used for the detection of the target protein. Blots were captured on X-ray films and quantified using Image J software (Version 1.52, Wayne Rasband, National Institutes of Health, Bethesda, MD, USA).

### 2.8. Quantitative RT-PCR Analysis

Fresh crushed leaves (approximately 100 mg) were used for gene expression analysis. According to the instruction given in the kit, total RNA was purified by Trizol reagent (Invitrogen, Carlsbad, CA, USA) after the extraction. Nearly 0.2 µg RNA was utilized for combining the first-strand cDNA with M-MLV reverse transcriptase (Promega, Madison, WI, USA) using an oligo (dT) primer. At that time, through gel electrophoresis, the quality of cDNA was examined, and the samples were kept at −80 °C for qRT PCR. For the study of gene expression, specific primers were designed (Table 1), and the fluorescent dye SYBR Green (Toyobo, Osaka, Japan) was used. After that, according to the manual, real-time PCR was performed using the real-time PCR Master Mix (Toyobo, Osaka, Japan). The housekeeping tubulin (TUB) gene worked as an internal control. The relative amount of the target gene expression was determined by the procedure of Chen et al. [45].

### 2.9. Statistical Analysis

Data were evaluated statistically using analysis of variance (ANOVA) and Tukey’s (post-hoc multiple comparison) test at *p* < 0.05 or *p* < 0.01 by SPSS 17.0 software (SPSS Inc., Chicago, IL, USA) for Windows. Data are presented as mean ± SE (*n* = 4).

## 3. Results

### 3.1. Screening of MeJA Concentration for Protection of Plants against Heat-Induced Oxidative Stress

The effect of different concentrations of MeJA on photosynthesis and growth was studied to assess the MeJA requirement of the crop under heat stress. Previous studies showed that MeJA plays a significant role in determining photosynthesis and growth of plants under stress [8,11]. We tested different concentrations of MeJA (5, 10, and 20 µM) to select the best concentration for maximum alleviation of heat stress. Application of 5 µM MeJA in comparison with heat stress, reduced H_2_O_2_ content and enhanced total protein content, *P*n, plant fresh weight, but the results were statistically similar to those recorded for the control. In contrast, the application of 20 µM MeJA notably increased H_2_O_2_ content, declined net photosynthesis, total protein content, and plant fresh weight compared to control under no stress and with heat stress (Table 2). Moreover, the results were different with the spraying of 10 µM MeJA. Application of 10 µM MeJA more effectively minimized H_2_O_2_ content by 76.6% and 48.6% under no stress compared to heat stress and control, respectively. The maximum reduction in H_2_O_2_ content by 81.4% and 59.2% was obtained with 10 µM MeJA under heat stress compared to heat stress and control, respectively (Table 2). Application of 10 µM MeJA also increased total protein, *P*n, and plant fresh weight by 24.1%, 23.8%, and 25.4% under no stress compared to control. Application of 10 µM MeJA to heat-stressed plants increased total protein content, *P*n, and plant fresh weight maximally by 35.6%, 37.5%, and 32.7%, respectively, in comparison with the control; differences were significantly greater with both concentrations of 5 and 20 µM MeJA under no stress and heat stress (Table 2). This showed that the requirements of plants were met with 10 µM MeJA under heat stress.

### 3.2. MeJA Enhanced Antioxidant Enzymes Activity and Reduced Oxidative Damage under Heat Stress

Earlier studies have shown that MeJA decreases the accumulation of oxidative stress by increasing the activity of antioxidant enzymes under stress [14]. However, reports on the effects of MeJA on antioxidant enzymes and the content of O_2_^−^, H_2_O_2_, and TBARS under heat stress in wheat are less studied. Therefore, we tested the effect of MeJA on O_2_^−^, H_2_O_2_, and TBARS content to determine the potential of MeJA in reducing oxidative stress. We found that plants under heat stress showed a rise in the content of H_2_O_2_ by more than two-fold and of TBARS by about three-fold compared to control plants. Treatment with MeJA decreased oxidative stress by reducing O_2_^−^, H_2_O_2_, and TBARS by 23.2%, 12.6%, and 12.1% under no stress and by 43.6%, 42.4%, and 50% in heat-stressed plants, respectively, compared to control (Table 3). The results showed that in comparison with heat stress, the application of MeJA to heat stress-treated plants had more significantly different effects than its application under no stress, decreasing O_2_^−^, H_2_O_2_, and TBARS by 59.6%, 76.3%, and 83.7%, respectively.

Heat stress augmented the activity of enzymatic antioxidants including CAT, SOD, APX, and GR by 21.0%, 43.4%, 40.1%, and 17.2%, respectively compared to control. Exogenous MeJA under no stress increased the activity of enzymatic antioxidants (CAT, SOD, APX, and GR) by 45.8% and 76.4%, 38.6% and 98.8%, 64.3% and 130.3%, and 24.6% and 46.1% as compared to heat stress and control, respectively. However, when heat-stressed plants were treated with MeJA, a maximum increase in the activity of CAT, SOD, APX, and GR was observed, corresponding to 54.1% and 86.5%, 50.1% and 115.3%, 73.2% and 142.8%, and 37.6% and 61.4% in comparison to heat-stress plants and control, respectively (Table 3). These results indicated that MeJA alleviation of oxidative stress was associated with increased activity of enzymatic antioxidants in wheat subjected to heat stress.

### 3.3. Pigments and Protein Content

Methyl jasmonate has been recognized as an important signal molecule that increases pigment and protein content in response to the different stresses [8,11]. Therefore, we studied the effects of MeJA on the pigment and protein content of plants under heat stress (Table 4). The results showed that heat stress treatment decreased Chl a, Chl b, Chl a-to-Chl b ratio, and total Chl content by 26.6%, 17.4%, 10.8%, and 25.1%, respectively, compared to control. The carotenoid content also decreased by 12.4% in heat-stressed plants. Application of MeJA under no stress enhanced the protein and pigment content significantly as compared to heat stress, but statistically the increase in Chl content was almost similar to what observed for the control under no stress. However, the application of MeJA in the presence of heat stress maximally enhanced Chl a, Chl b, Chl a-to-Chl b ratio, total Chl, and carotenoid content by 52.7% and 12.4%, 27.5% and 5.3%, 19.7% and 6.8%, 48.2% and 10.9%, and 63.3% and 43.0% compared to heat stress and control, respectively, and showed significantly more different results with respect to control, heat stress, and application of MeJA under no stress. Similarly, the protein content also increased upon MeJA application by 38.3% and 213.1% under heat stress in comparison to heat stress and control, respectively (Table 4).

### 3.4. Influence of MeJA on Chlorophyll a Fluorescence

**Figure 1:**
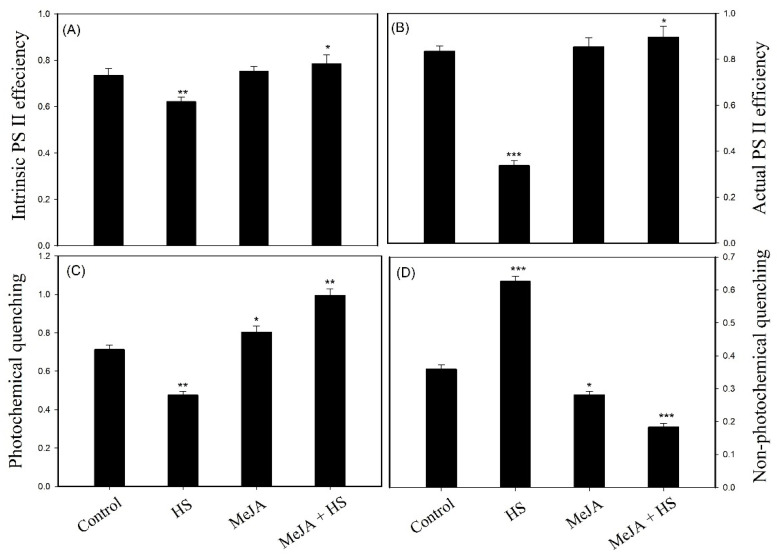
Intrinsic PS II efficiency (**A**), actual PS II efficiency (**B**), photochemical quenching (**C**), and non-photochemical quenching (**D**) in wheat leaves at 30 DAS. Plants were treated with MeJA (10 µM) at 42 °C (heat stress) or 25 °C (no stress). Data are presented as mean ± SE (*n* = 4). Significantly different values are marked with an asterisk between control and treatments (* *p* < 0.05, ** *p* < 0.01, *** *p* < 0.001), as determined by Tukey’s test. DAS, days after sowing; HS, heat stress; MeJA, methyl jasmonate.

Since previous studies have reported that MeJA repaired PS II in mustard and enhanced the photosynthetic efficiency of the system [15], we investigated the effect of MeJA on chlorophyll a fluorescence. Till date, there is no report available on the effect of MeJA on chlorophyll fluorescence under heat stress in wheat. Plants grown under heat stress exhibited reduced intrinsic PS II efficiency, actual PS II efficiency, and qP by 15.3%, 59.6%, and 33.3%, respectively, compared to control, and NPQ increased by 74.0%. Exogenous MeJA enhanced the above parameters significantly in comparison with heat-stressed plants, under no stress. However, a maximum increase in intrinsic PS II efficiency, actual PS II efficiency, and qP by 26.3% and 6.9%, 166.2% and 7.3%, and 109.4% and 39.6%, and a decline in NPQ by 70.7% and 49.0% was obtained with MeJA under heat stress, respectively, in comparison to heat stress and control (Figure 1A–D). These observations showed that MeJA increased the photosynthetic efficiency of PS II under heat stress.

**Figure 2:**
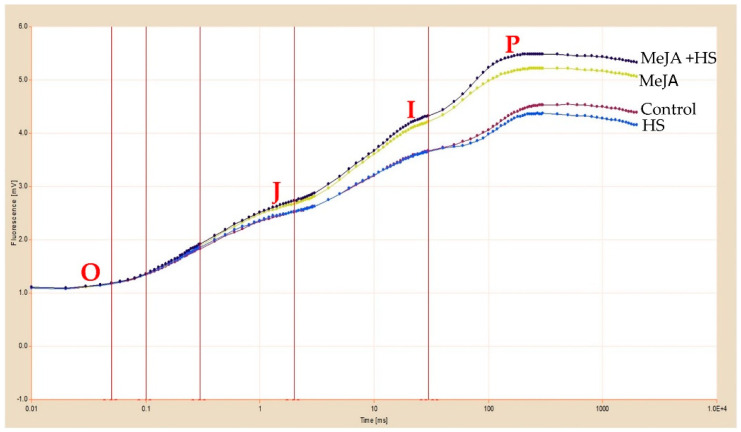
Chlorophyll fluorescence of OJIP curve in wheat leaves at 30 DAS. Plants were treated with MeJA (10 µM) at 42 °C (heat stress) or 25 °C (no stress). Data are presented as mean ± SE (*n* = 4). DAS, days after sowing; HS, heat stress; MeJA, methyl jasmonate.

It was interesting to note that MeJA was more effective under heat stress as compared to control. Possibly, efficient MeJA signaling is activated under heat stress. The chlorophyll fluorescence parameters varied under different treatments. Chlorophyll fluorescence increased from a minimum level (“O” or Fo) towards a maximum level (“*p*” or Fm). The results showed that in response to heat stress treatment, Fv/Fm and the diameter of leaves decreased to a very large degree, whereas for the MeJA-treated leaves, the results were reversed (Table 5). In heat-treated plants, both Fo and Fm decreased, and the O–J, J–I, and I–P phases showed lower amplitudes, in comparison to those in with MeJA-treated plants under control or heat stress. The OJIP curves were normalized at Fo to determine the changes in the fluorescence kinetics and show relative variable fluorescence vs. time on a logarithmic time ruler (Figure 2).

These results suggested that heat stress influenced chlorophyll fluorescence, as presented in OJIP curves (Figure 2). The fluorescence parameters Fo, Fm, Fv/Fm, and Fv/Fo decreased by 9.2%, 12.7%, 2.6%, and 4.9%, respectively, compared to control under heat stress. Nevertheless, the fluorescence parameters were higher with the application of MeJA to heat-stressed leaves by 31.5% and 19.4%, 65.4% and 44.3%, 7.5% and 4.7%, and 34.5% and 26.8%, respectively, compared to heat stress and control. Heat treatment decreased PI by 17.4%, but in MeJA-treated plants under heat stress it increased by 97.6 and 63.2% compared to heat stress and control, respectively. These results indicate the beneficial effect of MeJA compared to control and heat-stressed plants. The ratio of RC/ABS, which reveals RCs density of the PS II antenna chlorophyll, was higher in MeJA-treated plants in heat stress by 19.3%, which decreased by 9.3% under heat stress compared to control. The leaves treated with MeJA were more resistant to heat stress and reduced-heat adversities with respect to chlorophyll fluorescence. The area over the OJIP curve, between Fo and Fm, decreased upon heat stress treatment by 26.4% but increased by 55.7% and 14.5% in MeJA-treated leaves compared to heat stress and control, respectively (Table 5; Figure 2), again confirming the benefits of the MeJA treatment in heat-stressed wheat.

**Figure 3:**
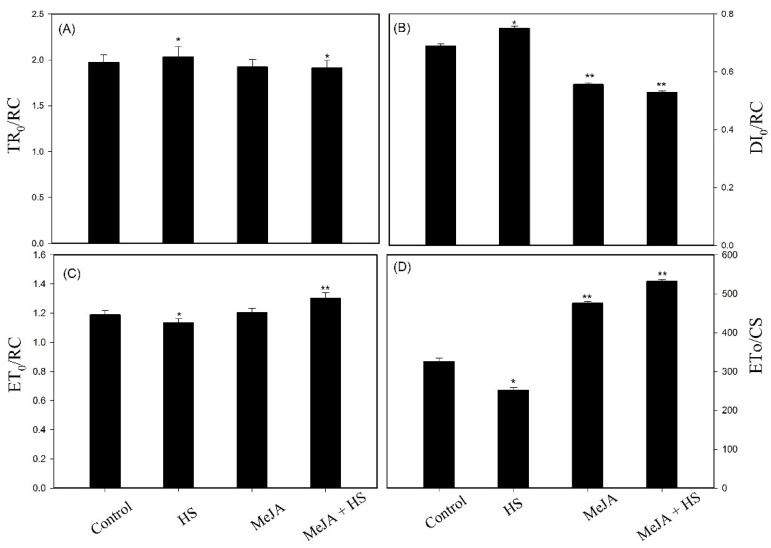
Photosynthetic parameters inferred from the JIP test study of chlorophyll fluorescence transients. Change of specific energy fluxes per PS II reaction center (RC) as (**A**) Trapping (TR_0_/RC), (**B**) Dissipation (DI_0_/RC), and (**C**) Electron transport (ET_0_/RC) and for (**D**) yields as quantum yields of electron transport (ET_o_/CS) in wheat leaves at 30 DAS. Plants were treated with MeJA (10 µM) at 42 °C (heat stress) or 25 °C (no stress). Data are presented as mean ± SE (*n* = 4). Significantly different values between control and treatments are marked with an asterisk (* *p* < 0.05, ** *p* < 0.01), as determined by Tukey’s test. DAS, days after sowing; HS, heat stress; MeJA, methyl jasmonate.

Additionally, other parameters were analyzed to specify energy fluxes (per RC) and yield, as shown in Figure 3A–D. Energy fluxes (per RC), i.e., TR_0_/RC (trapping) and DI_0_/RC (dissipation), were higher under heat stress. However, trapping and dissipation per RC were reduced significantly after the application of MeJA under heat stress (Figure 3A,B). There were noticeable effects on ET_0_/RC (electron transport flux per RC) and yield, indicated by ET_o_/CS (quantum yield of electron transport), after MeJA treatment under heat stress, as MeJA increased these values by 14.7% and 9.61% and 111.6% and 63.5% compared to heat stress and control, respectively (Figure 3C,D).

### 3.5. Impact of MeJA on Photosynthesis and Growth under Heat Stress or without Stress

Earlier studies have shown that MeJA regulates many aspects of plant development by involving photosynthetic characteristics [15,18]. We investigated the role of MeJA in improving the photosynthetic capacity in wheat. It has been reported that MeJA exerts a positive effect in response to abiotic stress [11,14]. To test the role of MeJA on photosynthesis and growth under heat stress, we treated wheat plants with 10 µM MeJA. Our results showed that heat stress-treated plants showed a decline in *P*n, Ci, and *g*s by 34.5%, 30.4%, and 26.4%, respectively, compared to control. Application of MeJA significantly increased these parameters (*P*n, Ci, and gs) under normal conditions compared to heat stress and control. However, MeJA treatment maximally benefitted the plants under heat stress and maximally alleviated the reduction in *P*n, Ci, and gs by 112.2% and 38.8%, 75.6% and 22.1%, and 65.3% and 21.6% compared to heat stress and control, respectively. These results verified that MeJA could increase the photosynthetic efficiency under heat stress. Heat stress reduced leaf area, plant fresh and dry mass by 43.3%, 47.4%, and 42.9%, respectively, compared to control. An individual dose of MeJA was effective in increasing leaf area, plant fresh and dry mass in the lack of heat stress. However, the maximal increase in the leaf area and plant fresh and dry mass by 32.6%, 34.6%, and 34.9% compared to control was noted with the application of MeJA to the heat-treated plants and proved more effective in lessening the effect of heat stress in wheat plants (Table 6).

### 3.6. Effect of MeJA on D1 Protein Content Abundance and Gene Expression Relevant to the Photosynthetic System

**Figure 4:**
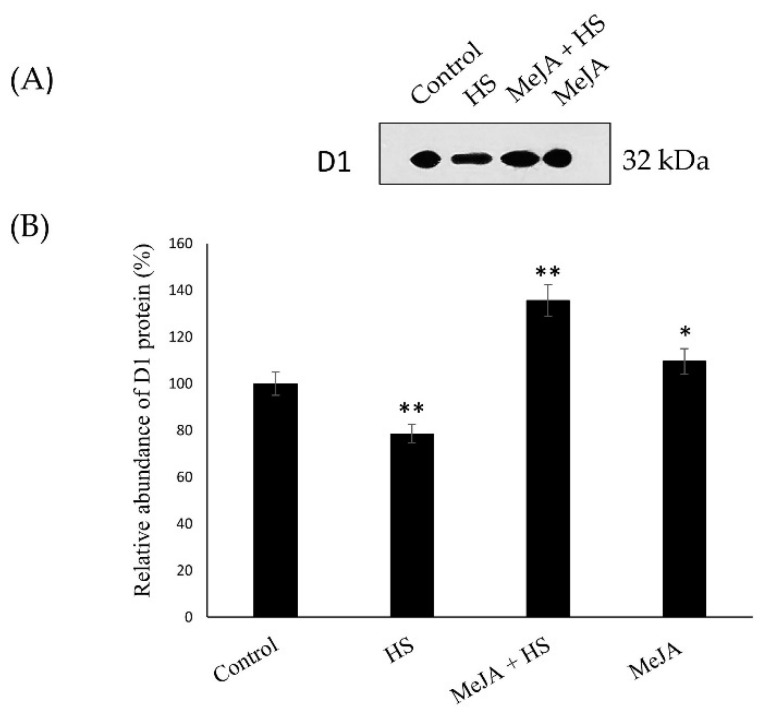
Immunoblot studies of thylakoid protein (D1) acquired from wheat leaves at 30 DAS. Plants were treated with MeJA (10 µM) at 42 °C (heat stress) or 25 °C (no stress). (**A**) Immunoblotting was performed with specific antibodies raised against the D1 protein. (**B**) Quantitative data for D1 protein in wheat leaves under heat stress after MeJA treatment. Results are presented relative to the respective controls (control, 100%, *n* = 4). Significantly different values are marked with an asterisk (* *p* < 0.05, ** *p* < 0.01). DAS, days after sowing; HS, heat stress; MeJA, methyl jasmonate.

The D1 protein constitutes the core of the PS II reaction center [2,5], and many studies have indicated that the PS II center in chloroplasts is the part that is the most easily damaged by environmental stresses [9]. Therefore, to identify the role of MeJA in the PS II system and in the maintenance of efficient turnover of D1 protein, the abundance of the PS II reaction center D1 protein in wheat leaves was analyzed. Our results suggest that the abundance of D1 protein in heat-treated leaves decreased significantly with respect to that in the control (Figure 4A,B). In contrast, with the application of MeJA, D1 protein abundance increased significantly compared to control and heat-stressed plants. The maximal increase in the abundance of D1 protein obtained with MeJA in heat-treated leaves was of 57.1% and 35.6% compared to heat-stressed and control leaves, respectively, and suggested that the recovery of D1 protein was effectively induced by MeJA in comparison with control. The original Immunoblot image is given in the Figure S1.

**Figure 5:**
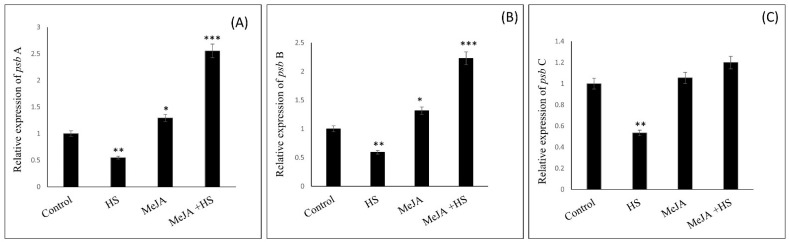
Relative expression of the genes (**A**) *psb*A; (**B**) *psb*B, and (**C**) *psb*C in wheat leaves at 30 DAS. Plants were treated with MeJA (10 µM) at 42 °C (heat stress) or 25 °C (no stress). Results are presented relative to the respective controls (control, 1). Data are presented as mean ± SE (*n* = 4). Significantly different values between control and treatments are marked with an asterisk (* *p* < 0.05, ** *p* < 0.01, *** *p* < 0.001), as determined by Tukey’s test. DAS, days after sowing; HS, heat stress; MeJA, methyl jasmonate.

Exogenous application of MeJA modulates the photosynthetic efficiency and the expression of PS II genes [15]. Therefore, we tested changes in the expression levels of PS II genes upon exogenous application of MeJA under heat stress. The photosynthetic system was examined to obtain the expression levels of *psb*A, *psb*B, and *psb*C genes, encoding D1 protein, CP47, and CP43, to explore the protective role of MeJA in wheat leaves in heat-stressed plants. We observed that the application of MeJA enhanced the expression of *psb*A and *psb*B significantly, but it did not affect *psb*C levels under normal conditions. Heat stress decreased the expression of genes compared to control. However, MeJA under heat stress strongly stimulated *psb*A and *psb*B expression, while the effect on *psb*C expression was lower compared to that on *psb*A and *psb*B (Figure 5A–C).

## 4. Discussion

Heat stress causes damage in plants’ photosynthetic apparatus, deterioration of leaf function, and reduction of yield. Degradation of chloroplasts and damage of PS II are important factors that affect photosynthesis. These aspects are currently the focus of research to understand the influence of stress on plants. Exogenous application of MeJA markedly regulates numerous key biochemical, physiological, and molecular processes in response to abiotic stress [14,46,47]. However, the effects of MeJA on PS II in wheat under heat stress are less known. Therefore, the present research was designed to acquire knowledge on how MeJA protects PS II and maintains the stability of D1 protein by regulating the expression of relevant genes during heat stress.

### 4.1. MeJA Increases Antioxidant System Activity to Mitigate the Oxidative Damage Induced by Heat Stress

Heat stress is one of the reasons for the accumulation of ROS such as H_2_O_2_ and superoxide ions, oxy-intermediates that cause cellular damage through the oxidation of lipids, proteins, and nucleic acids. The present study shows that exogenous MeJA markedly increased the activity of the antioxidant enzymes CAT and APX, which led to efficient detoxification of ROS and decreased lipid peroxidation in membranes under stress. Plants have developed complex defense systems as enzymatic antioxidants under heat stress; SOD changes superoxide ions into H_2_O_2_ and O_2_; then, CAT mainly changes H_2_O_2_ into H_2_O and O_2_ that is produced by photorespiration and β-oxidation of fatty acids, while GR and APX catalyze the transformation of H_2_O_2_ to H_2_O through dismutation [8]. Thus, we speculated that enhanced CAT, APX, GR, and SOD enzyme activities upon MeJA treatment played an effective role in the scavenging of O_2_^−^and H_2_O_2_. Lang et al. [14] reported that MeJA increased the activity of antioxidant enzymes and reversed the harmful effect of salt stress in *Glycyrrhiza uralensis*. Similarly, MeJA has been reported to increase the activity of POD and SOD in *Brassica napus* [46], of CAT, POD, and APX in *Citrus limon* [48], and of CAT, POX, and APX in *Fragaria× ananassa ‘Camarosa’* [49]. Remarkably, our results indicate that MeJA improved ROS-scavenging ability in wheat plants and exerted progressive effects on the improvement of plant resistance under heat stress.

### 4.2. MeJA Improved the Photosynthetic Efficiency under Heat Stress

The photosynthetic pigments Chl a and Chl b are the main pigments in leaves’ photosynthesis process, indicative of the physiological state of plants [32,50]. Both Chl a and Chl b absorb light energy, but only Chl a in the excited state transforms light energy into electrical energy and plays an important role in managing the stability of the light-harvesting complex related to PS II and in regulating the size of the photosynthetic antenna in plants [51]. Heat stress decreased the content of Chl a, Chl b, total Chl, carotenoid, and Chl a-to-Chl b ratio considerably (Table 4). The disturbance of Chl synthesis was linked to the decrease in Chl content in response to high temperature and cadmium stress [3,52]. Changes in the Chl a/b ratio are usually associated with changes in the size of the light-harvesting antenna of PS II. The results showed that the decrease of Chl content by heat stress was enhanced by MeJA. In addition, the Chl a/b ratio was higher under heat stress after MeJA treatment. Exogenous MeJA may act as a regulator inhibiting the disintegration of Chl molecules and protecting the photosynthetic antenna and PS II structure, thus enhancing the heat tolerance and photosynthetic efficacy of wheat through increasing the activity of antioxidant enzymes that scavenge ROS. These results are related to previous findings indicating that MeJA application protected the degradation of photosynthetic pigments under environmental stresses [8,14]. It was reported that jasmonate increased Chl a content, which is the direct photon donor to the RCs of both PS II and PS I systems [53]. Enhanced content of Chl a over Chl b helps sustain the photosynthetic capacity at a greater level, which promotes the accumulation of carbohydrates, after both jasmonate and MeJA treatments [15].

Increased Chl levels together with enhanced protein levels increased Fo in the plants (Table 4 and Table 5). These results are also supported by the chlorophyll fluorescence data, which showed a decrease in PS II quantum efficiency and yield (Table 5; Figure 1, Figure 2 and Figure 3). High temperature influences electron transport, which damages the function of PS II [41,54]. For the development of heat tolerance, wheat leaves were treated with MeJA, and then Chl fluorescence transients were examined by the JIP test (Figure 2; Table 5). Chl fluorescence kinetics transients provided additional data regarding the photochemical reaction in photosynthesis, mainly for the PS II donor side and the receptor side with RCs [41,55]. According to the JIP test, heat stress at 42 °C caused the impairment of the PS II donor side and an overreduction of the PS II acceptor side compared with those at optimal temperature (25 °C), resulting in a greater use of the excitation energy that decreased the stability of the photosystem [38] and altered the amplitudes O, J, I, and *p*_,_ as observed in the OJIP curve. A significant difference upon application of MeJA under heat stress was evident when examining the OJIP curve and the data of chlorophyll a fluorescence, shown in Figure 2 and Figure 3 and Table 5. This difference indicates that MeJA promotes heat resistance by protecting the PS II in wheat leaves through increasing quantum yield and efficiency. Heat stress lowers the stability of the PS II system [38], thereby leading to the decrease of PI. The performance index shows the photosynthetic capacity based on light absorption and is the most sensitive parameter in the OJIP curve. It comprises the maximum quantum yield of primary photochemistry, possibly related to the capability to reduce an electron acceptor at the end of PS I, and the RC/ ABS ratio or includes light absorption, trapping, and transfer excitation energy in electron transport [38,56,57]. The results showed that plants under heat stress did not adjust their light absorption and consumption, causing adverse effects on the RC. The present research shows that the application of MeJA had a beneficial impact on PI, which was higher compared to heat stress (Table 5). The application of MeJA influenced the trapped excitation and electron transport flux per RC, leading to normal levels. This proves that MeJA augmented the active RC and reduced the damage on RC in heat stress. In MeJA-treated plants under heat stress, PI and the RC/ABS ratio were higher than in the control (Table 5), suggesting improved stability of the PS II system due to comparatively higher stability of RCs, which contributed to higher PI in MeJA-treated plants exposed to heat stress. The results for PS II are consistent with those of studies on spermidine and nitric oxide application for the mitigation of PS II damage in tall fescue under heat stress [6,58].

The energy fluxes per RC, i.e., TR_0_/RC and DI_0_/RC, were higher, but ET_0_/RC and yield (ET_o_/CS) were reduced under heat stress (Figure 3). The results showed that the electron transportation efficiency and the function of Chl in PS II decreased under heat stress, but the application of MeJA remarkably improved ET_0_/RC and ET_o_/CS. Exogenous MeJA had a favorable effect on the acceptor and donor sides of PS II under heat stress. In addition, MeJA protected the RC of PS II and lessened the damage to the thylakoid protein complex organization by increasing Fv/Fm and qP. Exogenous MeJA protected the RCs, which were damaged under heat stress, and increased the size of light-harvesting antennas in the plants due to the increase of Chl molecules. Furthermore, the decrease in chlorophyll a fluorescence and the reduced qP are accountable for the decreased CO_2_ assimilation under heat stress. MeJA worked as regulator in the stomatal response and improved *P*n, Ci, gs, and the leaf area in plants under heat stress (Table 6), improving the efficiency of photosynthesis.

### 4.3. MeJA Increases D1 Protein Content and Gene Expression Relevant to the Photosynthetic System

Previous studies have shown that the PS II reaction center in chloroplasts, mainly, the D1 protein, was easily damaged by stresses [2,9]. Consistent with previous reports, the present study also showed that D1 protein, a sensitive component of the photosystem, was damaged by heat stress. It is important to maintain conformation stability of PS II RCs; therefore, D1 protein damage causes alteration of PS II RC conformation, electron transfer disruption, and destruction of PS II RCs [5,59,60]. The protection of the photosynthetic apparatus mainly depends on the stability of D1 under stress. Therefore, in the present study, wheat leaves were treated with MeJA under heat stress to examine the abundance of D1 protein and its function in PS II. It was possible that the damage of D1 protein and the repair of D1 protein under heat stress were reversible. However, in the presence of MeJA under heat stress, the level of D1 protein was much higher than in control and heat-stressed plants, indicating that MeJA treatment significantly reduced D1 protein degradation under heat stress by regulating enzymatic antioxidants. MeJA treatment not only reduced D1 protein damage or PS II function under heat stress, but also more efficiently restored D1 protein levels and PS II compared to control plants after heat stress, which was verified by the Fv/Fm results (Table 5). The application of MeJA to the heat-treated plants may increase D1 protein abundance more effectively and stimulate the assembly of PS II RCs through scavenging ROS by enhancing enzymatic antioxidants and increasing PS II activity, as PS II RC proteins are the main targets of ROS under stress responsible for the decrease in PS II function [60,61]. The mechanisms of the recovery of D1 protein levels and PS II function are not simple, as they are not just involved in the reducing the injured proteins but, also involved in the synthesis of new proteins, in particular in relation to *psb*A gene expression in chloroplasts. Therefore, further experiments were set up to analyze gene expression and determine the mechanisms influencing the stability and repair of D1 protein in the presence of MeJA under heat stress. The damaged D1 protein RCs under heat stress disturbed energy utilization and led to a reduction in CP43 and CP47 (*psb*C- and *psb*B-encoded proteins respectively), which are main antenna protein complexes of the PS II system [62,63]. Hence, the protection of D1 under heat stress is vital for the RC of PS II. The application of MeJA stimulated CP47 transcription and increased the function of RC to some extent. The protein D1 encoded by the *psb*A gene is a crucial element of the PS II system, and an assortment of cofactors involved in electron transfer and charge separation, are collectively organized in the PS II structure [64,65]. Our results showed that in the presence of MeJA, *psb*A gene expression increased significantly, which could be responsible for PS II stability under heat stress, as the *psb*A gene may participate in the restoration and renewal of D1 protein, damaged under heat stress. As shown in Table 3, the application of MeJA stimulated antioxidant enzyme activities, helped the scavenging of oxidative stress, upregulated the expression of genes coding for proteins involved in the PS II system (Figure 5), enhanced the removal of the negative effects of heat stress, and eventually increased the photosynthetic efficiency. Thus, MeJA protection of the photosystem under heat stress was reciprocated reflected in the increased abundance of D1 protein and overexpression of the *psb*A gene. In summary, MeJA reduced PS II core proteins degradation under heat stress, thereby contributing to heat tolerance in wheat plants.

## 5. Conclusions

The current study revealed that MeJA is efficient in the protection of the PS II complex under heat stress. Exogenous MeJA decreased heat-induced oxidative stress through increased activity of enzymatic antioxidants that protected the photosynthetic apparatus and enhanced chlorophyll content, chlorophyll fluorescence, and CO_2_ assimilation. These findings indicate that MeJA supply exerted a positive effect by maintaining the stability of chloroplast D1 protein in the PS II complex and enhancing the expression of relevant genes. This study suggests that MeJA can be utilized to enhance PS II efficiency and overall protection of photosynthesis in adverse climatic conditions.

## Figures and Tables

**Table 1:** Primer sequences and data used for RT-PCR analyses.

Gene	Encoded Polypeptide	Gene ID	Forward(F) /Reverse (R)	Primer Sequences (5′–3′)	Size (bp)
*psb*A	D1 protein	7095419	F R	GTATTTATTATCGCCTTCATCG AGGACGCATACCCAAACG	284
*psb*B	CP47	7095420	F R	TAGGCGTAACGGTGGA AACATCTCGGAACAAGG	254
*psb*C	CP43	7095484	F R	TAATACGGCTTATCCGAGTGAGTTT TCTTGCCAAGGTTGTATGTCTTT	288

Zhang et al. [6]; Chen et al. [45].

**Table 2:** Content of H_2_O_2_ and total protein, net photosynthesis, and plant fresh weight of wheat leaves 30 DAS. Plants were treated with MeJA (5, 10, and 20 µM) at 42 °C (heat stress) or 25 °C (no stress). Data are presented as mean ± SE (*n* = 4). Significantly different values between control and treatments are marked with an asterisk (* *p* < 0.05, ** *p* < 0.01, *** *p* < 0.001), determined by Tukey’s test. DAS, days after sowing; FW, fresh weight; HS, heat stress; H_2_O_2_, hydrogen peroxide; MeJA, methyl jasmonate.

Treatments	H_2_O_2_ Content (nmol g^−1^ Leaf FW)	Total Protein (mg g^−1^ Leaf FW)	Net Photosynthesis (µmol CO_2_ m^−2^ s^−1^)	Plant Fresh Weight (g Plant^−1^)
Control	15.2 ± 0.89	8.70 ± 0.86	16.8 ± 0.82	3.85 ± 0.12
HS	33.4 ± 0.98 **	3.60 ± 0.42 **	10.9 ± 0.56 **	1.55 ± 0.06 **
5 µM MeJA	10.6 ± 0.76 *	9.60 ± 0.88	18.1 ± 0.93	4.07 ± 0.11
10 µM MeJA	07.8 ± 0.56 **	10.8 ± 1.21 *	20.8 ± 0.96 **	4.83 ± 0.19 *
20 µM MeJA	19.3 ± 0.84 *	5.84 ± 0.65 **	11.2 ± 0.64 **	2.83 ± 0.08 *
5 µM MeJA + HS	17.2 ± 0.73	6.65 ± 0.73 *	13.1 ± 0.71 *	3.79 ± 0.09
10 µM MeJA + HS	06.2 ± 0.66 **	11.8 ± 1.42 **	23.1 ± 0.97 ***	5.11 ± 0.22 **
20 µM MeJA+ HS	40.1 ± 1.02 **	2.73 ± 0.13 **	09.2 ± 0.54 ***	1.29 ± 0.05 **

**Table 3:** Production rate of superoxide radicals (O_2_^−^), content of H_2_O_2_, TBARS, activity of CAT, SOD, APX, and GR in wheat leaves at 30 DAS. Plants were treated with MeJA (10 µM) at 42 °C (heat stress) or 25 °C (no stress). Data are presented as mean ± SE (*n* = 4). Significantly different values between control and treatments are marked with an asterisk (* *p* < 0.05, ** *p* < 0.01, *** *p* < 0.001), as determined by Tukey’s test. APX, ascorbate peroxidase; CAT, catalase; DAS, days after sowing; FW, fresh weight; GR, glutathione reductase; HS, heat stress; H_2_O_2_, hydrogen peroxide; MeJA, methyl jasmonate; SOD, superoxide dismutase; TBARS, thiobarbituric acid reactive substances.

Treatments				
Parameters	Control	HS	MeJA	MeJA + HS
Production rate of O_2_^−^ (µmol g FW^−1^ min^−1^)	0.801 ± 0.05	1.118 ± 0.080 **	0.611 ± 0.060 *	0.451 ± 0.02 **
H_2_O_2_ content (nmol g^−1^ leaf FW)	35.60 ± 1.60	86.80 ± 02.40 ***	31.10 ± 01.1 *	20.50 ± 0.09 **
TBARS content (nmol g^−1^ leaf FW)	08.2 ± 0.12	25.3 ± 0.19 **	07.2 ± 0.09 *	04.1 ± 0.07 **
CAT activity (U mg^−1^ protein min^−1^)	119 ± 3.70	144 ± 4.00 *	210 ± 4.30 **	222 ± 5.10 **
SOD activity (U mg^−1^ protein min^−1^)	05.34 ± 0.08	07.66 ± 0.11 *	10.62 ± 0.18 **	11.5 ± 0.21 **
APX activity (U mg^−1^ protein min^−1^)	1.12 ± 0.04	1.57 ± 0.09 *	2.58 ± 0.11 **	2.72 ± 0.11 ***
GR activity (U mg^−1^ protein min^−1^)	0.197 ± 0.005	0.231 ± 0.008 *	0.288 ± 0.009 **	0.318 ± 0.01 ***

**Table 4:** Content of Chl a, Chl b, Chl (a/b) ratio, total Chl, and carotenoids in wheat leaves at 30 DAS. Plants were treated with MeJA (10 µM) at 42 °C (heat stress) or 25 °C (no stress). Data are presented as mean ± SE (*n* = 4). Significantly different values between control and treatments are marked with an asterisk (* *p* < 0.05, ** *p* < 0.01, *** *p* < 0.001), as determined by Tukey’s test. Chl, chlorophyll; DAS, days after sowing; FW, fresh weight; HS, heat stress; MeJA, methyl jasmonate.

Treatments				
Parameters	Control	HS	MeJA	MeJA + HS
Chl a (mg g^−1^ Leaf FW)	1.71 ± 0.06	1.26 ± 0.04 **	1.75 ± 0.06 *	1.92 ± 0.08 **
Chl b (mg g^−1^ Leaf FW)	0.45 ± 0.01	0.37 ± 0.01 **	0.46 ± 0.02	0.47 ± 0.04 *
Chl (a/b)	3.79 ± 1.70	3.38 ± 1.56 **	3.80 ± 1.73	4.05 ± 1.77 **
Total chl (mg g^−1^ Leaf FW)	2.15 ± 0.07	1.62 ± 0.04 **	2.21 ± 0.07 *	2.39 ± 0.08 **
Carotenoids (mg g^−1^ Leaf FW)	0.44 ± 0.01	0.38 ± 0.01 **	0.49 ± 0.02 *	0.63 ± 0.05 ***
Total protein (mg g^−1^ Leaf FW)	08.60 ± 1.18	03.80 ± 1.09 **	10.90 ± 1.39 **	11.90 ± 1.42 **

**Table 5:** Chl a fluorescence measurement in wheat leaves at 30 DAS. Plants were treated with MeJA (10 µM) at 42 °C (heat stress) or 25 °C (no stress). Minimal fluorescence (Fo), maximal fluorescence (Fm), maximal variable fluorescence (Fv), and Fv/Fm ratio, where Fv = (Fm−Fo), reaction center-to-PS II antenna absorption ratio (RC/ABS), performance index (PI), and Area, region above the chlorophyll fluorescence OJIP curve between Fo and Fm. Data are presented as mean ± SE (*n* = 4). Significantly different values between control and treatments are marked with an asterisk (* *p* < 0.05, ** *p* < 0.01, *** *p* < 0.001), as determined by Tukey’s test. DAS, days after sowing; HS, heat stress; MeJA, methyl jasmonate.

Treatments				
Parameters	Control	HS	MeJA	MeJA + HS
Fo	206 ± 03.80	187 ± 03.50 **	241 ± 04.10 **	246 ± 04.40 **
Fm	935 ± 07.30	816 ± 06.20 *	1258 ± 08.10 **	1350 ± 08.50 ***
Fv/Fo	2.863 ± 0.018	2.709 ± 0.011 *	3.445 ± 0.021 **	3.639 ± 0.026 **
Fv/Fm	0.780 ± 0.04	0.760 ± 0.04 *	0.808 ± 0.05 **	0.817 ± 0.05 **
RC/ABS	0.831 ± 0.07	0.754 ± 0.04 *	0.956 ± 0.08 **	0.992 ± 0.08 **
PI	1.675 ± 0.014	1.383 ± 0.009 *	2.423 ± 0.021 **	2.734 ± 0.023 ***
Area	21,744 ± 505	15,989 ± 421 *	23,100 ± 539	24,907 ± 611 *

**Table 6:** Net photosynthesis, intercellular CO_2_ concentration, stomatal conductance, leaf area, plant fresh and dry weight of wheat leaves at 30 DAS. Plants were treated with MeJA (10 µM) at 42 °C (heat stress) or 25 °C (no stress). Data are presented as mean ± SE (*n* = 4). Significantly different values between control and treatments are marked with an asterisk (* *p* < 0.05, ** *p* < 0.01, *** *p* < 0.001), determined by Tukey’s test. Chl, chlorophyll; DAS, days after sowing; FW, fresh weight; HS, heat stress; MeJA, methyl jasmonate.

Treatments				
Parameters	Control	HS	MeJA	MeJA + HS
Net photosynthesis (µmol CO_2_ m^−2^ s^−1^)	16.2 ± 0.91	10.6 ± 0.52 ***	20.5 ± 095 **	22.5 ± 0.99 ***
Intercellular CO_2_ concentration (µmol CO_2_ mol^−1^)	230 ± 9.1	160 ± 7.3 **	258 ± 10.3 **	281 ± 11.1 **
Stomatal conductance (mmol H_2_O m^−2^ s^−1^)	310 ± 12.3	228 ± 8.7 **	345 ± 13.5 **	377 ± 14.1 **
Leaf area (cm^2^ Plant^−1^)	106 ± 4.1	60.1 ± 2.9 ***	123 ± 4.3 **	140.6 ± 4.9 ***
Plant fresh weight (g Plant^−1^)	5.37 ± 0.09	2.82 ± 0.05 **	6.01 ± 0.10 *	7.23 ± 0.11 **
Plant dry weight (g Plant^−1^)	0.808 ± 0.04	0.461 ± 0.01 **	0.897 ± 0.06 *	1.090 ± 0.09 **

## Data Availability

Data is contained within the article and supplementary material.

## Supplementary Materials

The following are available online at https://www.mdpi.com/article/10.3390/antiox10081216/s1, Figure S1: Immunoblot analyses of thylakoid protein (D1) obtained from wheat leaves 30 days after sowing. Plants were treated with MeJA (10 µM) at 42 °C (heat stress) or 25 °C (no stress). Immunoblotting was performed with specific antibodies raised against the D1 protein.
