# Supplementary material for: Methyl Jasmonate Protects the PS II System by Maintaining the Stability of Chloroplast D1 Protein and Accelerating Enzymatic Antioxidants in Heat-Stressed Wheat Plants
Source: Antioxidants (Basel). 2021 Jul 28;10(8):1216. doi: 10.3390/antiox10081216 (PMC8388886; doi:10.3390/antiox10081216)
Supplement: Supplementary file 1 [file antioxidants-10-01216-s001.zip › antioxidants-1279921-supplementary.pdf]

antioxidants

MDPI

Article

Methyl Jasmonate Protects the PS II System by Maintaining the Stability of Chloroplast D1 Protein and Accelerating Enzymatic Antioxidants in Heat-Stressed Wheat Plants

Mehar Fatma [1], Noushina Iqbal [2], Zebus Sehar [1], Mohammed Nasser Alyemeni [3], Prashant Kaushik [4], Nafees A. Khan [1,*] and Parvaiz Ahmad [3,*]

[1] Plant Physiology and Biochemistry Laboratory, Department of Botany, Aligarh Muslim University, Aligarh 202002, India; meharfatma30@gmail.com (M.F.); seharzebus5779@gmail.com (Z.S.)
[2] Department of Botany, School of Chemical and Life Sciences, Jamia Hamdard, New Delhi 110062, India; naushina.iqbal@gmail.com
[3] Botany and Microbiology Department, College of Science, King Saud University, Riyadh 11451, Saudi Arabia; mnalyemeni@gmail.com
[4] Kikugawa Research Station, Yokohama Ueki, 2265, Kamo, Kikugawa City, Shizuoka 439-0031, Japan; kaushik.prashant@yokohamaueki.co.jp
* Correspondence: na.khan.bt@amu.ac.in or naf9.amu@gmail.com (N.A.K.); Pahmad@ksu.edu.sa or parvaizbot@yahoo.com (P.A.)

Supplementary Figure S1

Supporting Information File for Western blotting

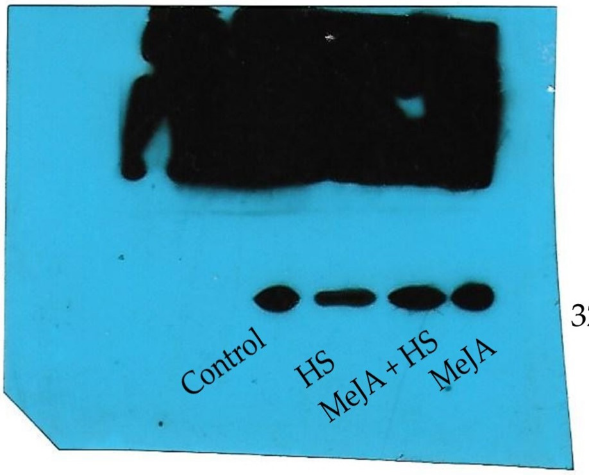

D1 protein
32kDa (M.W.)

Figure S1. Immunoblot analyses of thylakoid protein (D1) obtained from wheat leaves at 30 days after sowing. Plants were treated with MeJA (10 μM) in presence (42 °C) or absence (25 °C) of heat stress. Immunoblotting were done using specific antibodies raised against the D1 protein. HS, heat stress; MeJA, methyl jasmonate.
